# Exploiting Microbial Polysaccharides for Biosorption of Trace Elements in Aqueous Environments—Scope for Expansion via Nanomaterial Intervention

**DOI:** 10.3390/polym9120721

**Published:** 2017-12-16

**Authors:** Manikandan Muthu, Hui-Fen Wu, Judy Gopal, Iyyakkannu Sivanesan, Sechul Chun

**Affiliations:** 1 Department of Environmental Health Science, Konkuk University, Seoul 143-701, Korea; bhagatmani@gmail.com (M.M.); jejudy777@gmail.com (J.G.); 2 Department of Chemistry, National Sun Yat-Sen University, Kaohsiung 80424, Taiwan; hwu@faculty.nsysu.edu.tw; 3 Department of Bioresources and Food Science, Konkuk University, 1, Hwayang-dong, Gwangjin-gu, Seoul 143-701, Korea; isivanesan@gmail.com

**Keywords:** biosorption, biogenic polysaccharide, heavy metal, aqueous environments

## Abstract

With pollution sounding high alarms all around us, there is an immediate necessity for remediation. In most cases, the remediation measures require further remediation—the anti-pollutants themselves cause pollution. In this correspondence, the search deepens towards natural biogenic components that can be used for bioremediation. Polysaccharide and biosorption have been themes in discussion for quite some time, where a slow decline in the enthusiasm in this area has been observed. This review revisits the importance of using polysaccharide based materials for biosorption. The need for polysaccharide-based nanocomposites, which hold better promise for greater deliverables, is emphasized as a means of rejuvenating the future perspectives in this area of application.

## 1. Introduction

Heavy metal pollution occurs directly by effluent outfalls from industries, refineries and waste treatment plants and indirectly via contaminants entering the water supply from soil/ground water systems and from the atmosphere via rain water [1]. Among toxic substances reaching hazardous levels in aquatic systems, the primary concern is from heavy metals [2]. Exposure to heavy metals has been linked with developmental retardation, various cancers, kidney damage and even death in the case of contact with very high concentrations. The anthropogenic sources of heavy metals include wastes from chemical factories, mine drainage, battery manufacturing, leather industries, fertilizer industries, and pigment manufacturing industries as well as leachates from landfills and contaminated ground water owing to hazardous waste sites [3]. The European Economic Community has issued a directive on pollution caused by heavy metals, discharged into the aquatic environment [4]. Moreover, the World Health Organization has announced a separate list of heavy metals of immediate concern, which includes but is not limited to: aluminum, chromium, manganese, iron, cobalt, nickel, copper, zinc, cadmium, mercury and lead.

Current decontamination methods involve ion-exchange technologies and precipitation of the cations in an inert form [4,5,6,7]. However, the limitation of using these systems is the utilization of products that themselves could become contaminants [8,9,10]. Recent research work on the removal of heavy metals focused on the fabrication of materials that demonstrate increased affinity, capacity and selectivity for target metals [11,12]. This is where the employment of microorganisms as biosorbents of heavy metals gains prominence. This technology is an inexpensive alternative compared to other conventional methods used in heavy metal bioremediation [9,13,14].

The advantage of biosorption is that it uses biomass and industrial wastes that are cheap and abundant [15]. Biosorbents can gather the heavy metals from the solution by manipulating the properties of a biosorbent, or upon desorption during the regeneration cycle of these biosorbents [16,17,18]. Algae, bacteria and fungi are well established for their successful ability to mop up toxic heavy metals (Cd^2+^, Hg^2+^, Zn^2+^, and Pb^2+^), precious metals (Au^3+^, Pd^2+^, and Ag^+^), base heavy metals (Co^2+^, Ni^2+^, and Cu^2+^) and radionuclides (U^6+^ and Th^4+^) from their respective environments [19,20,21,22,23,24,25]. Thus far, different types of microbial biomass have been used for the clean-up of industrial effluents, including algae, bacteria, fungi and yeasts [26], or even extracellular materials such as extracellular polysaccharides (EPS) [27,28].

Bacterial cells find a way to protect themselves from the infiltration of toxic metal ions by covering its peripheral surface with a shield of EPS. EPS is a defense strategy by bacteria to keep itself from the external environment and for establishing biofilm communities on solid substrates. Structural and compositional makeup of EPS thus renders it favorable for the sequestration of metal ions. Because of this property, microbial polymers have been comprehensively investigated for biosorption of heavy metal contamination. These microbial polymers can exist either as attached capsular polysaccharides (CPS) or as slime upon microbial surfaces [29]. Compositionally, they are often seen to be polymerized hexose sugar moieties and exist as either homo or heteropolysaccharides [29]. Bacterial homopolysaccharides include dextran, levan, curdlan and heteropolysaccharides comprise of xanthan, alginate and hyaluronan and more so. Intracellular synthesis of polysaccharides (homo or hetero) is a comparatively complex process, and proceeds via intracellular assembly, after which it is transported outside the cell [29,30,31,32]. The current review gives a brief introduction of the current achievements made through biosorption, and the advances made through the integration of biosorption. The mechanisms of biosorption and modes of biosorption at work are discussed. The emphasis on enlarging the future perspective of this field through incorporation of nanomaterials has been elaborated and discussed in the concluding remarks.

## 2. Mechanism of Biosorption

The mechanism of biosorption has been described in detail by Ahalya et al. [33]. Biosorption of metal ions onto microorganisms occurs through a combination of several metal-binding mechanisms. These mechanisms include: physical adsorption, ion exchange, complexation and precipitation. Physical adsorption involves Van der Waal forces (electrostatic interaction) between metal ions in the solution and the cell wall of the microbes. For example, *Zoogloea ramigera*, *Chlorella vulgaris* and *S. saprophyticus* mediate their copper and lead biosorption using this mechanism [34]. Complexation involves metal ion removal from an aqueous solution by complex formation of metals on the cell surface following interaction between metal ions and active groups. Metal ions are biosorbed or complexed by carboxyl groups that are components of microbial polysaccharides. Aksu et al. [35] reported copper biosorption onto *Zoogloea ramigera* and *Chlorella vulgaris* via both adsorption and the formation of coordination bonds between metals and the carboxyl and amino groups of cell wall components. Similar results on biosorption of electroplating heavy metals by some basidiomycetes fungi has been reported by Javaid and Bajwa [36]. The third mechanism is through ion exchange where the polysaccharides that exist on cell walls of microorganisms possess ions, such as K^+^, Na^+^, Ca^2+^ and Mg^2+^, with metal ions, resulting in metal ion uptake [26,37]. The last mechanism is precipitation, which happens dependent or independent of cellular metabolism. Metal ion removal from aqueous solutions is often associated with the active defense system of microorganisms, favoring the precipitation process [38].

Biosorption is rendered possible by two independent modes, the first is via metal binding by either whole organisms such as algae [39,40] or bacteria [41,42]. The second mode is operational through molecules such as biopolymers [43,44]. Biopolymers possess hydroxyl, carbonyl, carboxyl, sulfhydryl, thioether, sulfonate, amine, imine, amide, imidazole, phosphonate, and phosphodiester functional groups. These functional groups exhibit a natural tendency to bind metals. Table 1 presents a few predominant bacteria and their metal affinity with their specific polysaccharides and the respective functional groups that orchestrate this affinity.

## 3. Microbial Polysaccharides—Applications and Relevance in Biosorption

Polysaccharides are significant components of biological systems that have been extracted and put to extensive use, and are also known as biopolymers. Dextran (DeX), pullulan (PuL), cellulose (CeL), chitosan (CS), hyaluronic acid (HA), alginate (ALG) and many more [45,46,47,48,49,50] are those that come under the nomenclature of biopolymers. For instance, HA is microbial in origin and is a main component of the extracellular matrix. It consists of *N*-acetyl-d-glucosamine and d-glucuronic acid residues. CeL, which is a main component of plant cells and few bacteria too, is said to be the most abundant polysaccharide in nature. It consists of a homopolymer of β(1 → 4) linked d-glucose. CS, the cationic polysaccharide, is composed of β(1 → 4) linked 2-amino-deoxy-d-glucan, resulting from the deacetylation of *N*-acetyl-d-glucosamine of chitin (CT). Recently, the extracellular polysaccharide, succinoglycan and its production by Sinorhizobium, Agrobacterium and other soil bacteria have gained importance [50]. One of the well-known EPS producers are the rhizobia, which excrete large amounts of polysaccharides into the rhizosphere as well as when grown in pure cultures [51]. Polysaccharides are characteristically highly abundant in nature, renewable, nontoxic, intrinsically biodegradable and relatively cheap. Furthermore, they possess functional groups such as hydroxyl, amino (e.g., CS), and carboxylic acids (e.g., HA and ALG). These functional groups can be further modified too. With these added features, polysaccharide-based biomaterials have various other extended applications such as drug delivery carriers, cell-encapsulating biomaterials, tissue engineering scaffolds and for regenerative medicine [52,53,54,55,56].

### 3.1. Four Different Strategies of Microbial EPS Mediated Trace Element Biosorption

In a recent review, Gupta et al. (2017) [57] have elaborated on the different strategies through which biosorption by microbial polysaccharides operates. Each mode is differentiated based on the source of the EPS: be it from pure cultures (homogeneous consortial EPS), or mixed cultures (heterogeneous consortium), dead cells (Dead biomass) or immobilized cells (immobilized EPS), the common thread is their ability to orchestrate biosorption of heavy metals.

#### 3.1.1. Metal Biosorption Following Homogeneous Consortial EPS

Examples of this mode of biosorption in action is reported in the case of a methylotrophic bacterium *Methylobacterium organophilum* which has been reported to exhibit copper and lead ion removal nonspecifically within half an hour of reaction incubation [58]. Another Gram-negative bacterium *Herminiimonas arsenicoxydans* was found to scavenge arsenic ions through EPS interaction. [59,60]. Betaproteobacteria *Thiomonas* sp. CB2 was also reported to trap arsenic ions in its EPS [61]. Marine micro-alga associated *Halomonas* sp. was reported to chelate calcium, silicate, iron, magnesium and aluminum metal ions [62]. Strains of *Shewenella oneidensis*, *Agrobacterium species*, and *Rhizobium tropici* have also been found to produce polysaccharides which displayed substantial cadmium ion adsorption. *Rhizobium radiobacter* EPS was confirmed for its biosorption capacity of lead and zinc ions [63]. EPS like succinoglycan and galactoglucan from another *Rhizobium* species *Sinorhizobium meliloti*, illustrated arsenic and mercury ion resistance. [64]. Copper ions were reported to be preferentially adsorbed over zinc ions by *Klebsiella* sp. **J1** EPS [65]. The EPS of haloalkaliphilic *Bacillus* sp. has been demonstrated for its potential application in treatment of lead contaminated waters, as reported by earlier workers [66]. An EPS extracted and purified from *Arthrobacter ps-5* showed efficient biosorption of Cu^2+^, Pb^2+^ and Cr^2+^ [67]. Chitin and chitosan are extracted from fungal mycelia, and workers [68] have demonstrated that chitin from *Cunninghamella elegans* could conduct copper, lead and iron biosorption in aqueous solution.

#### 3.1.2. Metal Biosorption Following Heterogeneous Consortial EPS

Mixed culture bacterial consortia orchestrating heavy metal biosorption are well demonstrated too. Liu Y. et al. reported that activated sludge mixed cultures could reduce almost 85–95% of zinc, copper and chromium [69]. In addition, other Gram-negative bacterial consortia could reduce 75–85% of initial metal ions including zinc, lead, chromium, copper, cadmium and cobalt efficiently in less than two hours [70]. EPS of microbial consortium isolated from hydrocarbon contaminated water source was observed to reduce Cd^2+^; Zn^2+^ and Cu^2+^ [71].

#### 3.1.3. Metal Biosorption by Dead Biomass EPS

Dead biomass EPS have also been well established for biosorption, for example, EPS of dead biomass of floc forming bacterium *Ochrobactrum anthropi* removed cadmium ions along with other toxic metals [72]. Live and dead biomass bound EPS of three different bacteria, *Bacillus cereus*, *Bacillus pumilus* and *Pantoea agglomerans*, were approved for their chromium ion biosorption capacity [73].

#### 3.1.4. Metal Biosorption Using Immobilized EPS

Advancements in immobilization techniques verify that attachment of bacterial cells to solid surfaces stimulates EPS production without altering the specific growth rate [74]. This was demonstrated in *Chryseomonas luteola* immobilized in alginate bead along with its EPS for examining cadmium, cobalt, nickel and copper ion adsorption and *Paenibacillus Polymyxa* EPS immobilized in agar beads was evaluated for uptake of lead ions [75].

Polysaccharides are thus considered as green and sustainable resources for remediation of heavy metal polluted aqueous environments. These inputs from biogenic polysaccharides suggest the preeminence of microbial polysaccharides in accomplishing trace element biosorption. Tailored polysaccharides [76,77,78,79], which have contributed greatly to various applications, are promising integrations. Therefore, the incorporation of tailored polysaccharides with much higher specificity and/or metal biosorption capacity would prove to be more promising. The future of these biogenic polysaccharides for biosorption has also been further enhanced through nano based biopolysaccharide complexes.

## 4. Biogenic Polysaccharide Nanomaterial Based Biosorption

Nanotechnology has embarked and revolutionized almost every aspect of science. Nano based systems have thoroughly boosted water purification systems via refined filtration mechanisms by carbon nanotube (CNT) based membranes [80,81,82,83]; advanced detoxification of menacing pollutants using zero-valent iron NPs; detection of impurities and pathogens by nanosensors; photocatalytic degradation of water pollutants by titanium dioxide NPs; nanoporous polymers, nanoporous zeolites, and attapulgite clays for water treatment; and magnetic NPs for water purification and remediation [84]. In addition, nanotechnology has innovated filters and membranes that are made from different nanomaterials such as CNTs, dendrimers, nanoporous ceramics (clays), nanofibers, zeolites and nanosponges. These nanomaterials have a clear edge over the existing technologies owing to their high porosity, active metal binding sites, small sizes, regeneration after exhausting and speed of contaminant removal [85,86].

**Figure 1:**
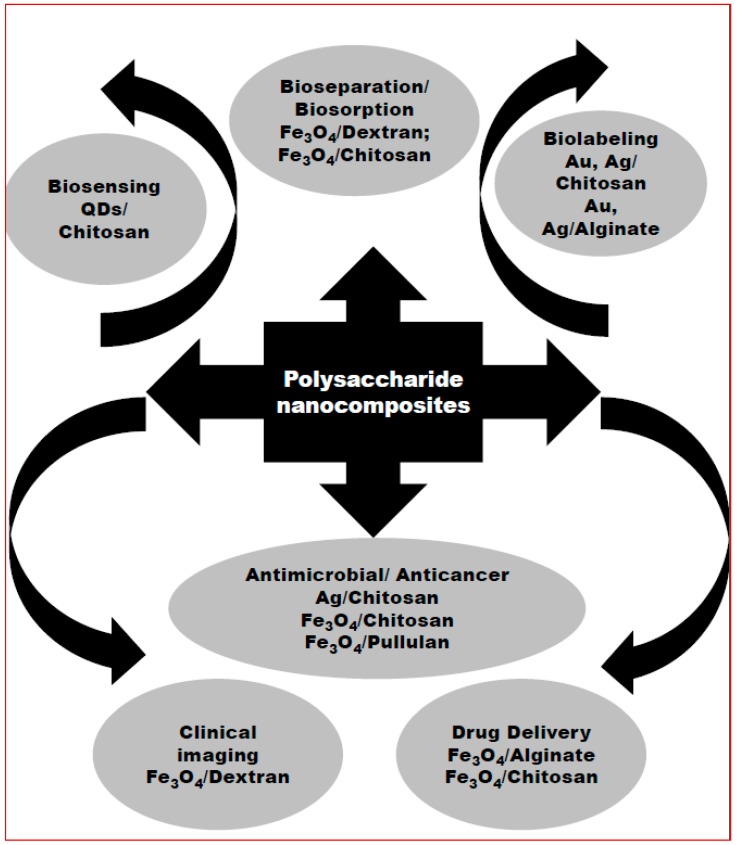
Overview of current scenario of polysaccharide nanocomposite applications.

The term “nanocomposite” is a relatively new terminology where nano materials are integrated with proteins, lipids, sugars, polymers and others. Polysaccharide-nanomaterial composites have their own impact through their diverse applications. Many polysaccharide-based magnetic nanocomposites, such as magnetite (Fe_3_O_4_)–dextran, Fe_3_O_4_–chitosan, Fe_3_O_4_–alginate, Fe_3_O_4_–heparin, Fe_3_O_4_–pullulan acetate, Fe_3_O_4_–starch, Fe_3_O_4_–κ–carrageenan, and maghemite (γ-Fe_3_O_4_)–dextan/sucrose, have been demonstrated in applications such as bioseparation and purification [87,88], bioassays and sensors [89,90,91], biolabeling and imaging [92,93], cancer hyperthermia [94,95], cardiovascular therapies [96] and drug delivery [97,98] (Figure 1).

The term hydrocolloid is used on long chain polymers (polysaccharides and proteins) characterized by their ability to form viscous dispersions and/or gels when dispersed in water. Due to the large number of hydroxyl (–OH) groups they show marked affinity for binding water molecules rendering them hydrophilic. Further, they produce an intermediate dispersion which is neither a true solution nor a suspension, and exhibit properties of a colloid. Polysaccharides can also be brought under this nomenclature. It is with respect to this same property of possessing highly enhanced affinity for water molecules that the use of polysaccharides for biosorption of metal ions from water faces a huge limitation. This limitation can be overcome through the fabrication of nanointegrated polysaccharide complexes, where the nanomaterials used could offer the hydrophobic aspect to the polysaccharide in order to offset its high water absorption and retention.

Silica nanosols have been demonstrated for biocers that possess mechanical stability as well as porosity that are typical of the silicate matrix with the algal components ability for biosorption of heavy metals. Thirteen different microalgae and macroalgae have been used for biosorption of nickel at concentrations as less as 3 mg/L and even mixtures of different heavy metals (Cr, Ni, Cu, Pb) [99]. Nanocomposites based on chitin and chitosan (CS) are also making incredible progress. Chitin and CS have been widely studied in the removal of heavy metal ions from wastewater [100]. Fierro et al. [101] reported that the CS bead-immobilized algal system with *Scenedesmus* sp. could effectively take up phosphate and nitrate from water much better than the conventional cell system. The biosynthesis of silver nanoparticles using polysaccharide-based bioflocculant using *Streptomyces* sp. MBRC-91 has been reported by Manivasagan et al. [102]. The biosynthesized silver nanoparticles exhibited strong antibacterial activity in sewage water leading to a new avenue in the wastewater treatment.

Recently, polyamide-6/chitosan blended nanofibers were successfully produced by electrospinning [103]. Polyamide-6 is a biodegradable, biocompatible and synthetic polymer that has strong mechanical and physical properties [104]. It has been reported that electrospinning of polyamide-6/chitosan blended nanofibers combine the advantages of polyamide-6 together with those of chitosan that has high hydrophilicity, biocompatibility, biodegradability, antibacterial and antifungal activities. Thus, blending of these materials could revolutionize the versatility of membrane applications [105]. More recently, in 2015, Wang et al. have reported the successful integration of the “modern age material” graphene with chitosan for disinfection of water [106]. Table 2 gives an overview of the polysaccharides that have been so far used in the preparation of bionanocomposites.

## 5. Future Perspective of Biogenic Polysaccharide Nanocompositesfor Biosorption

**Figure 2:**
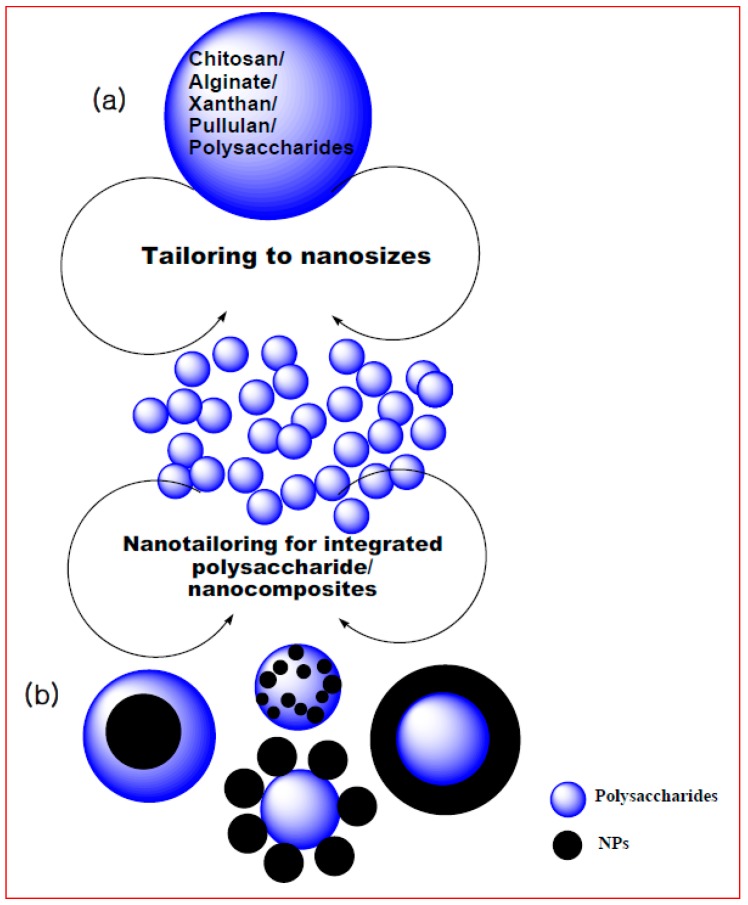
Future perspective of biosorption realized through: (**a**) nanosizing polysaccharides; and (**b**) integrating polysaccharides with nanoparticles (NPs) forming polysaccharide nanocomposites.

Polysaccharide application for biosorption has held its own edge over the conventional players in this application. However, biogenic polysaccharides have much more to offer; it is time to dig deep into the properties offered by these amazing natural components synthesized freely in nature. Instead of anthropogenic synthesis of nanomaterials for clearing pollution, which themselves result in pollution in indirect ways [107,108], it is time we shift our focus to harnessing naturally available safe and inherent components such as these. The future of polysaccharides for biosorption lies in two vital nanointegration domains (Figure 2). The first domain is via nanosizing the polysaccharides, in creating nanosized chitosan, cellulose nanofibers, alginate nanobeads, and pullulan nanofibrils; by doing so, we are combining the unique inherent properties of polysaccharides with the concurrent properties that are attached to nanosized materials. These properties include greater surface area to volume ratio over larger particles, higher available absorption sites and the versatility that flows with nanostructures. This would lead to the actual transition of this technique of biosorption to “nanosorption”.

The second domain towards a breakthrough in the future of biosorption is through the development of nanocomposites, consisting of polysaccharide moieties integrated with reputed and well established nanoparticle systems. The nanocomposites combine the properties of the polysaccharide with those of the nano systems, which this leads to a cutting edge development in the nanosorption technology. Few examples of nanocomposites and their accomplishments have been mentioned in the sections above. However, to make major breakthroughs, it is vital that not just few but various permutation combinations need to be attempted and implemented. MNPs, which are excellent nanosystems that also enable easy recovery with the help of an external magnet, QDs, dendrimers and carbon nanomaterials, including the family of CNTs, fullerenes, and graphene, would be attractive options for polysaccharide-nanocomposites. This review observed the sort of saturation in the field of nanosorption this decade; polysaccharide-nanocomposites will prompt more action and activity in this area and make room for accomplishments and for a wider scope and future for polysaccharide based biosorption research.

## Figures and Tables

**Table 1:** Specificity of bacterial polysaccharides for metals.

Strain	Metal Specificity	Biopolymers Involved
*Escherichia coli* K-12	Sr^2+^, Ce^3+^, Pr^2+^, ${\mathrm{UO}}_{2}^{2+}$, Sc^3+^, La^3+^, Co^2+^, Hg^2+^, Pb^2+^, Cu^2+^	*N*-acetylglucosamineNacetylmuramic acid crosslinked sugar residues, Lipopolysaccharide (LPS), EPS
*Pseudomonas aeruginosa*	La^3+^	LPS
*Acinetobacter lwoffii* RAG-1 TF1-35	${\mathrm{UO}}_{2}^{2+}$	apoemulsan
*Thiobacillus ferrooxidans* TF1-35	${\mathrm{UO}}_{2}^{2+}$	LPS
*Bacillus licheniformis*	Cr^3+^	γ-glutamyl capsular polymer
*Enterobacter aerogenes*	Cd^2+^	EPS
*Zooglea ramigera*	${\mathrm{UO}}_{2}^{2+}$, Cu^2+^, Cd^2+^	Zooglan
*Xanthomonas campestris*	Cu^2+^	Xanthan
*Cunninghamella elegans*	Cu^2+^, Pb^2+^	Chitosan
*Chlorella vulgaris*	Cu^2+^, Pb^2+^	EPS

**Table 2:** List of microbial biogenic polysaccharides used as bionanocomposites.

Polysaccharide	Source	Ionic Nature	Active Functional Group
**Gellan**	*Sphingomonas elodea*	Anionic	OH
**Dextran**	*Leuconostoc mesenteroides*, *Lactobacillus sps and Steptococcus mutans*	Neutral	OH
**Pullulan**	*Aureobasidium pullulans*	Neutral	OH
**Cellulose**	*Aerobacter*, *Acetobacter*, *Achromobacter*, *Agrobacterium*, *Alcaligenes*, *Azotobacter*, *Pseudomonas*, *Rhizobium and Sarcina*	Neutral	OH
**Hyaluronic acid**	*Streptococcal sps* and *Bacillus subtilis*	Anionic	OH
**Curdlan**	*Alcaligenes faecalis var myxogenes 10C3*, *Agrobacterium sps*	Cationic	OH, COO^−^
**Alginate**	*Pseudomonas sps and Azotobacter vinelandii*	Anionic	OH, COO^−^
**Chitosan**	*Cunninghamella elegans*, Fungal cells walls	Cationic	OH, COO^−^
**Xanthan**	*Xanthomonas campestris*	Anionic	OH
**Zooglan**	*Zoogloea ramigera*	Anionic	OH
**Succinoglycan**	*Agrobacterium sps*, *Rhizobium sps*, *Rhizobium meliloti*, *Alcaligenes faecalis*	Anionic	OH
